# *Rounding Out* the Understanding of ACD Toxicity with the Discovery of Cyclic Forms of Actin Oligomers

**DOI:** 10.3390/ijms22020718

**Published:** 2021-01-13

**Authors:** Harper Smith, Nick Pinkerton, David B. Heisler, Elena Kudryashova, Aaron R. Hall, Kelly R. Karch, Andrew Norris, Vicki Wysocki, Marcos Sotomayor, Emil Reisler, Dimitrios Vavylonis, Dmitri S. Kudryashov

**Affiliations:** 1 Department of Chemistry and Biochemistry, The Ohio State University, Columbus, OH 43210, USA; smith.12510@buckeyemail.osu.edu (H.S.); pinkerton.56@buckeyemail.osu.edu (N.P.); David.Heisler@UTSouthwestern.edu (D.B.H.); kudryashova.1@osu.edu (E.K.); karch.22@osu.edu (K.R.K.); norris.702@osu.edu (A.N.); wysocki.11@osu.edu (V.W.); sotomayor.8@osu.edu (M.S.); 2 Biophysics Graduate Program, The Ohio State University, Columbus, OH 43210, USA; 3 Ohio State Biochemistry Program, The Ohio State University, Columbus, OH 43210, USA; 4 Infectious Diseases Institute, The Ohio State University, Columbus, OH 43210, USA; 5 Department of Physics, Lehigh University, Bethlehem, PA 18015, USA; aah217@lehigh.edu (A.R.H.); Vavylonis@lehigh.edu (D.V.); 6 Department of Chemistry and Biochemistry, University of California, Los Angeles, CA 90095, USA; reisler@mbi.ucla.edu

**Keywords:** bacterial toxins, actin, actin cross-linking domain, ACD, MARTX, isopeptide bond, multivalent interactions, formin, Ena/VASP, polymerization, mass spectrometry, molecular dynamics

## Abstract

Actin is an essential element of both innate and adaptive immune systems and can aid in motility and translocation of bacterial pathogens, making it an attractive target for bacterial toxins. Pathogenic *Vibrio* and *Aeromonas* genera deliver actin cross-linking domain (ACD) toxin into the cytoplasm of the host cell to poison actin regulation and promptly induce cell rounding. At early stages of toxicity, ACD covalently cross-links actin monomers into oligomers (AOs) that bind through multivalent interactions and potently inhibit several families of actin assembly proteins. At advanced toxicity stages, we found that the terminal protomers of linear AOs can get linked together by ACD to produce cyclic AOs. When tested against formins and Ena/VASP, linear and cyclic AOs exhibit similar inhibitory potential, which for the cyclic AOs is reduced in the presence of profilin. In coarse-grained molecular dynamics simulations, profilin and WH2-motif binding sites on actin subunits remain exposed in modeled AOs of both geometries. We speculate, therefore, that the reduced toxicity of cyclic AOs is due to their reduced configurational entropy. A characteristic feature of cyclic AOs is that, in contrast to the linear forms, they cannot be straightened to form filaments (e.g., through stabilization by cofilin), which makes them less susceptible to neutralization by the host cell.

## 1. Introduction

Owing to its involvement in many aspects of host immune response, the actin cytoskeleton serves as a common target for pathogenic bacteria. By delivering proteinaceous actin-targeting toxins, bacteria can hijack actin polymerization machinery to disrupt the delicate balance between monomeric and filamentous actin, thereby disorganizing the actin cytoskeleton and compromising host defenses. Actin-targeting bacterial toxins work either indirectly, by acting on signaling cascades, or directly, by physically modifying actin. Toxin effectors that directly modify actin can be classified into three groups based on their mechanism: (1) cleavage between T351 and F352 to inhibit actin polymerization [1]; (2) ADP-ribosylation of T148 or R177 that results in bulk aggregation [2] or complete disassembly of the actin cytoskeleton [3], respectively; (3) intermolecular covalent cross-linking of actin monomers at K50 and E270 by the actin cross-linking domain (ACD) to generate actin oligomers (reviewed in [4]) with potent inhibitory activity towards several families of actin binding proteins [5,6].

ACD is an effector protein produced by gram-negative aquatic and human pathogens. ACD is delivered into the host cytoplasm via Type VI secretion system as part of the valine-glycine repeat protein G1 (VgrG1) toxin [7], or through the Type I secretion system, along with other domains of the multifunctional auto-processing repeats-in-toxin (MARTX) [8]. Once in the cytoplasm, ACD uses ATP to catalyze the formation of an isopeptide bond between K50 (located in the DNase-binding, or D-loop) and E270 (in the hydrophobic plug, or H-plug) of separate actin monomers [9], generating actin oligomers (AOs) of varying length [10]. Since a covalently linked H-plug and D-loop cannot normally contribute to filament contacts, AOs do not form filaments unless stabilized by phalloidin or cofilin [11].

It was initially suggested that cross-linked actin oligomers are passively toxic, i.e., gradually destroy the actin cytoskeleton due to bulk accumulation of non-functional oligomeric species [10]. Later, we showed that AOs exert potent toxicity in small doses (2–6% of total cytoplasmic actin) by binding to critical, low-abundance actin assembly proteins [5,6]. Accordingly, linear AOs disrupt formin-controlled polymerization of actin, WASP-activated branching by the Arp2/3 complex, Ena-accelerated filament growth, and Spire-mediated nucleation, when present in nanomolar concentrations [5,6]. All these actin assembly factors contain multiple sites that can bind either (i) directly to actin (e.g., through short ~17-amino acid WASP-homology motifs 2 (WH2-domains) [12], or (ii) via polyproline (PP)-rich motifs to actin-profilin complexes [13]. Therefore, the potency of AOs stems from their multivalent interaction with multiple G-actin binding domains within a single protein or complex. Thus, the inhibition of formin mDia1 by AOs in the presence of profilin showed reduced efficiency when PP-rich motifs are removed [6]. More broadly, apparent affinities of AOs for actin organizers scale proportionally to the number of G-actin binding domains: formin and Ena (_app_K_d_ ~2–6 nM) are better inhibited by AOs than N-WASP and Spire (_app_K_d_ ~8–14 nm) [5].

While analyzing western blot images of actin cross-linked in living cells at advanced stages of ACD toxicity, we discovered an unusual cross-linking array (“anomalous” oligomers) that overlaps with the regular pattern of AOs. This finding implies that the toxin may employ different mechanisms of toxicity at late stages or when present at higher doses. Here, we used electron microscopy, polymerization assays with and without filament stabilizers, and mass spectrometry to establish that the unusual pattern is due to cyclic forms of AOs. Coarse-grained molecular dynamics (MD) simulations predicted the most populated structures for both AO types and confirmed that cross-linked geometries typically expose binding sites for profilin (~65–75% exposure) and for the WH2-domain (~96–99% exposure). In agreement with stronger configurational constraints as compared to the linear forms, cyclic AOs inhibit formin and Ena to a moderately reduced extent in the presence of profilin. In contrast to the linear AOs, however, the cyclic oligomers cannot be incorporated into filaments by cofilin or the actin-stabilizing drug phalloidin, suggesting their higher resistance to neutralization and possibly higher potential to disrupt the actin cytoskeleton.

## 2. Results and Discussion

### 2.1. Discovery of a New Form of ACD-Cross-Linked Actin Oligomers

**Figure 1:**
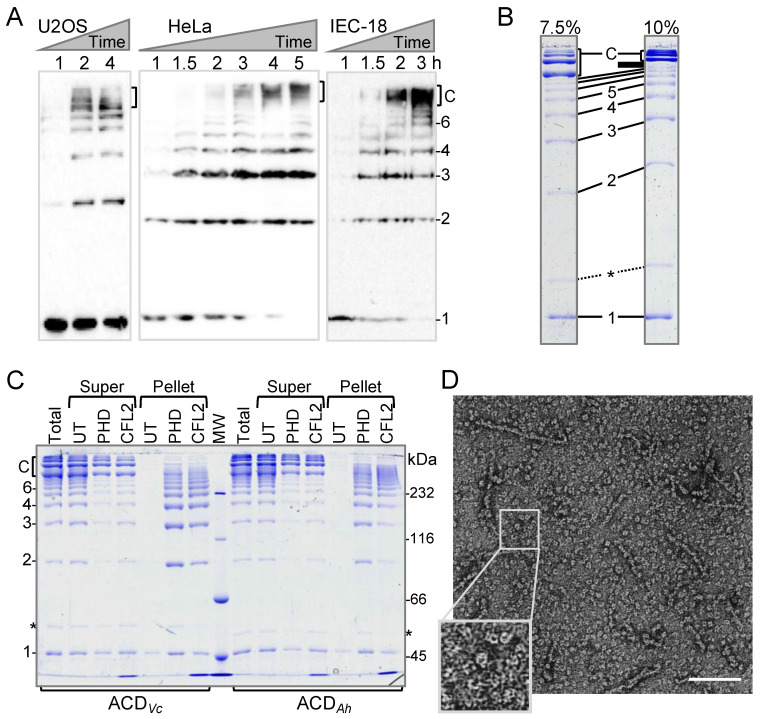
Actin cross-linking domain (ACD)-catalyzed formation of linear and cyclic covalently cross-linked actin oligomers. (**A**) Accumulation of ACD-cross-linked actin species upon cytoplasmic delivery of ACD was monitored by anti-actin immunoblotting of whole cell lysates. 1—actin monomers, 2–6—number of subunits in linear actin oligomers, C—cyclic actin oligomers accumulating at advanced stages of ACD toxicity (2–4 h). The blot lines were not cropped. (**B**,**C**) In vitro cross-linked actin oligomers were either spun untreated (UT) or following the pre-incubation with either phalloidin (PHD) or cofilin 2 (CFL2). Total—total cross-linking reactions before ultracentrifugation; Super—supernatant fractions; Pellet—pellet fractions; 1–6 and C—as in (**A**); asterisks denote ACDs. Identical samples were resolved on 7.5 and 10% SDS-polyacrylamide gels (**B**) to demonstrate the unusual mobility of circular actin oligomers in comparison to linear oligomers. Full-size uncropped gel images are shown in the Supplementary Figure S1A. (**D**) A representative transmission electron microscopy image of the entire population of ACD-cross-linked actin oligomers. The boxed area is enlarged for clarity. The scale bar is 100 nm.

ACD catalyzes covalent cross-linking of residue K50 on one actin protomer to E270 on another actin [9,11]. Consequent addition of monomers or even cross-linked oligomers [14] results in accumulation of oligomers of various lengths. These actin oligomers (AOs) thus normally appear on SDS-polyacrylamide gels as a series of bands, which progressively decrease in intensity at higher molecular weights. We noticed that, at advanced stages of cell treatment by ACD, the regular pattern of AOs is interrupted by cross-linked species of unusual mobility at the top of the immunoblots (Figure 1A). We were able to reproduce this new pattern in vitro, which resolved the AOs as an intercalating array of oligomers with different mobility and spacing pattern between the bands (Figure 1B). We also found that the production of unusual AOs is favored at high ACD to actin ratios (e.g., 1:100; Figure 1C) when ACDs from *Vibrio cholerae* or *Aeromonas hydrophila* MARTX toxins were used. Interestingly, the migration pattern of the anomalous bands on SDS-polyacrylamide gels—relative to the “normal” oligomers—depended on the acrylamide percentage (Figure 1B; Supplementary Figure S1A), suggesting that their mobility was not strictly size-dependent.

ACD operates by catalyzing a glutamyl-phosphate intermediate at actin’s E270, which becomes covalently linked by amide bond to K50 of another actin molecule [9]. No other actin residues can be cross-linked by ACD [9,11], suggesting that the bands of abnormal mobility should also be of the same chemical nature. To reconcile the identical chemical nature with the abnormal migration pattern on SDS-polyacrylamide gels, we hypothesized that the new AOs represent cyclic forms of the oligomers, whose terminal subunits are covalently linked together via the same K50 to E270 isopeptide bond. Electron microscopy analysis of the entire population of actin oligomers supported this hypothesis by revealing that the oligomers have a horseshoe- or donut-like configuration (Figure 1D).

Experimental data and mathematical modeling of AO-inhibited polymerization [6] suggest that linear forms of AOs can be incorporated into filaments assembled from non-cross-linked actin, albeit ineffectively. In this case, the contacts weakened by cross-linking are compensated by polymorphism of actin filaments [15] under the condition of abundance of normal actin. The oligomers do not polymerize on their own but can be induced to polymerize by filament stabilizers (such as phalloidin) or by proteins that heavily rearrange F-actin structure (i.e., by ADF/cofilin proteins) [11]. Phalloidin does not substantially change F-actin, but acts as a molecular glue by binding at the interface between three protomers [16,17]. Thereby, phalloidin is known to stabilize F-actin assembly from impaired actin species [18,19,20].

Cofilin, unlike phalloidin, changes the twist and structure of the actin filament by weakening both longitudinal and lateral actin contacts [21,22], but compensates for the diminished contacts with new actin-cofilin contacts by forming a bridge between adjacent protomers [23]. Particularly, cofilin reduces the functional load on the D-loop by releasing it from longitudinal inter-protomer contacts and leading to its disorder [24,25] and increased susceptibility to proteolysis [26]. Notably, if the abnormal oligomers are indeed covalently linked into a ring, they cannot be straightened, and both phalloidin- and cofilin-mediated mechanisms of stabilization should fail to rescue their polymerization. Therefore, we tested whether the anomalous AOs can be incorporated into actin filaments. Ultracentrifugation of heavily cross-linked actin did not bring either of the AO forms into the pellet (Figure 1C, “UT”). In contrast, addition of phalloidin (“PHD”) or the muscle isoform of human cofilin (“CFL2”) promoted the formation of filaments from regular but not the abnormal AOs (Figure 1C).

**Figure 2:**
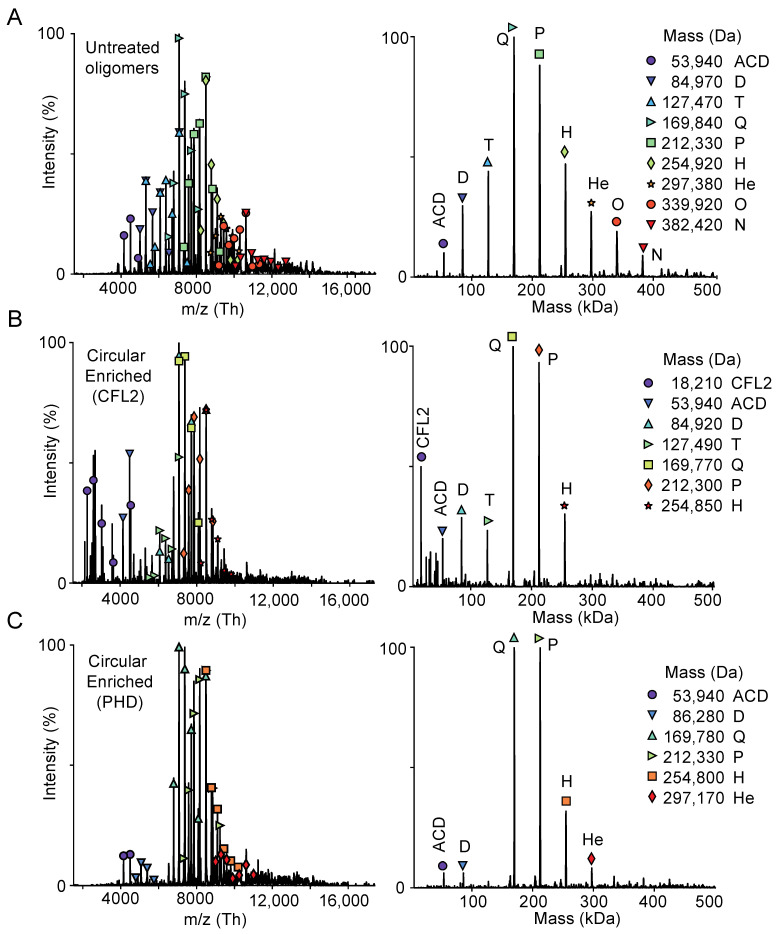
Mass spectrometry analysis of ACD-cross-linked actin oligomers. Oligomeric state of actin oligomers was assessed by mass spectrometry (MS). Samples subjected to MS were similar to those shown on Figure 1C (supernatant fractions): the entire population of untreated actin oligomers (**A**) and circular oligomers enriched by the removal of linear actin oligomers pelleted in the presence of either cofilin 2 (CFL2; (**B**)) or phalloidin (PHD; (**C**)). Panels on the left display an averaged raw spectrum; (the m/z axis denotes the measured mass to charge ratio of analyte ions in the sample); panels on the right display the deconvolution of the raw data as calculated by UniDec software. Masses were calculated using UniDec. M—monomer; D—dimer; T—trimer; Q—tetramer; P—pentamer; H—hexamer; He—Heptamer; O—Octamer; N—Nonamer. Comparison of the expected and observed masses is given in the Supplementary Table S1.

Pelleting the phalloidin- or cofilin-stabilized linear AOs allowed us to enrich the population of abnormal AOs in the supernatant and determine their size by mass spectrometry (Figure 2, Supplementary Table S1). We found that the predominant species have masses corresponding to tetramers, pentamers, and hexamers, despite their migration on SDS-polyacrylamide gels at the level of linear deca- or higher order oligomers. Overall, several lines of evidence combine to unambiguously suggest that the new form of AOs, produced at the advanced stages of toxicity or at high concentration of ACD, are indeed cyclic oligomers, in which the terminal subunits are covalently linked to each other.

### 2.2. Structural Basis of Actin Oligomer Incorporation into F-Actin

**Figure 3:**
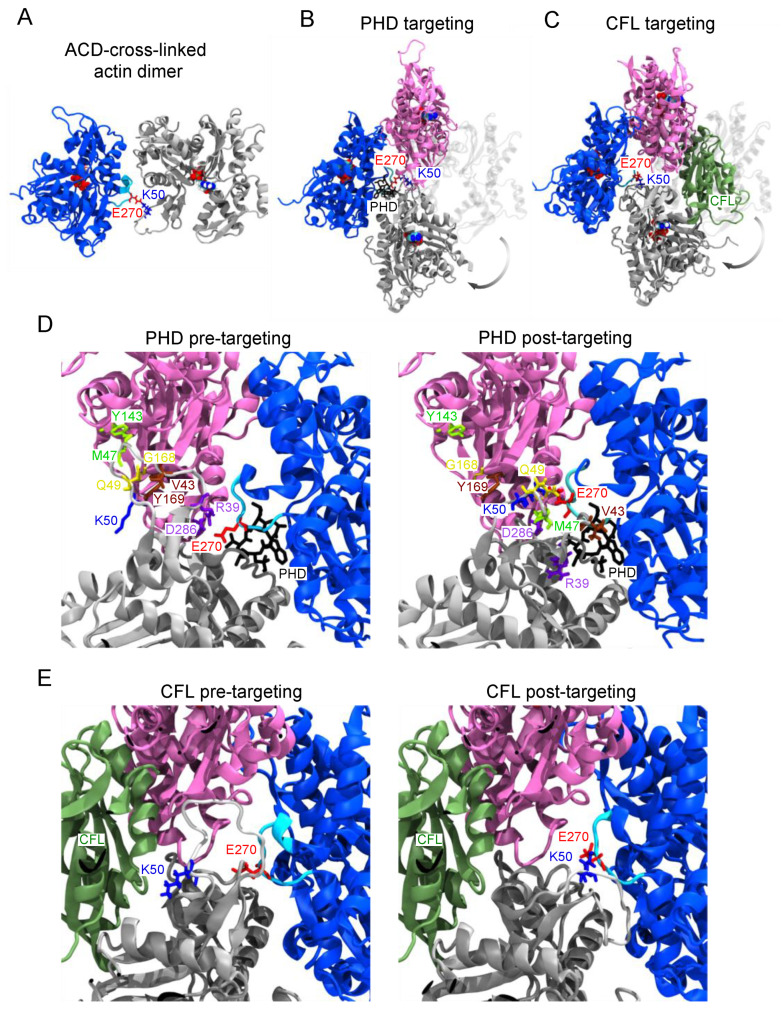
F-actin contacts disrupted by ACD cross-linking revealed by targeted molecular dynamics simulations (TMD). TMD was performed from the ACD-cross-linked dimer crystal structure (PDB: 3CJC) shown in (**A**) toward the configuration of F-actin trimers extracted from cryo-electron microscopy structures of (**B**) phalloidin-actin (PDB: 6T1Y); or (**C**) cofilin-actin (PDB: 6VAO). Arrows on (**B**,**C**) indicate the transition of the grey actin protomer upon targeting. Phalloidin (black; **B**) and cofilin (green; **C**) are shown only for clarity and were not part of the simulations. Spheres indicate the ATP molecule. K50 (blue sticks) in the D-loop (light gray) of one actin subunit (grey) is covalently cross-linked to E270 (red sticks) in the hydrophobic plug (cyan) of another actin subunit (blue) on (**B**,**C**,**D**, left, **E**, left). Enlarged structures in (**D**) show contact residues in the D-loop (light gray) and the longitudinally adjacent actin protomer (pink) in matching colors. In phalloidin-stabilized actin (**D**, left), interacting residues are colored in pairs V43/Y169 (brown), M47/Y143 (light green), N49/G168 (yellow), and R39/D286 (purple). All initially interacting pairs have separated by the post-targeting structure extracted from the final time of the simulation (**D**, right). In analogy to (**D**), none of the same contacts exist in cofilin-actin (PDB: 6VAO) shown on (**E**).

Despite evidence that linear AOs can integrate into F-actin with support from phalloidin or cofilin, the ACD-cross-linked residues are not in proximity; the side-chain nitrogen of K50 and the oxygen of E270 are separated by 18.5 Å in the cryo-electron microscopy structure of phalloidin-stabilized actin [16] (PDB: 6T1Y). Bringing these residues together via isopeptide bond should disrupt the inter-protomer interfaces in which the D-loop and H-plug participate. To understand how linear AOs could be accommodated in F-actin when rescued by phalloidin and cofilin, we employed atomistic targeted molecular dynamics (TMD) [27]. The starting arrangement of the subunits in the oligomer (Figure 3A) was taken from a crystal structure of an AO dimer determined in complex with gelsolin segment 1 (GS1) and DNase I (PDB: 3CJC) [11].

In the simulation, one protomer from the AO dimer was aligned with an F-actin trimer, and forces were applied to the ACD-cross-linked protomers to bring them to F-like orientation. In this process, two major transitions had to be accomplished: (1) the D-loop had to be rearranged in a sterically favorable way; and (2) both subunits from the AO-dimer had to flatten from G- to F-like conformation [28]. Since phalloidin-actin and cofilactin have a different characteristic twist, both transitions were examined (Figure 3B,C; Supplementary Movies S1 and S2). No steering forces were applied to the D-loop or H-plug, so they could rearrange passively throughout the transition.

A comparison of the cross-linked dimer with F-actin structures does not immediately suggest how an AO dimer could be incorporated into a filament. This alignment (Figure 3B) shows that an ~180° rotation is necessary to bring the cross-linked dimer into F-like configuration. Since the D-loop [29] and H-plug [30] impart considerable flexibility to the K50/E270 cross-link, the simulation was not meant to reproduce the actual transition, but to reveal the final AOs’ conformation in the filamentous state. We identified four longitudinal and one lateral contacts contributed by the D-loop in the cryo-electron microscopy structure [16] of phalloidin-actin (Figure 3D, left). At the end of the simulation (Figure 3D, right), all four longitudinal contacts are broken in the phalloidin F-state cross-linked by ACD. At this stage, the D-loop has settled neatly between ACD-cross-linked protomers while the H-plug remained close to its pre-targeting conformation.

In contrast to bare F-actin, the contribution of the D-loop to longitudinal contacts in cofilactin are minor and limited to R62 [25]. Since cofilactin is substantially stabilized by cofilin bridges, the contacts holding cofilactin together may not differ substantially, regardless if the filaments are assembled from AOs or unperturbed actin. To test this hypothesis in MD simulations, we had to rebuild the D-loop (Figure 3E, left) missing in all recent reconstructions of cofilactin. Forcing the transition to the highly twisted actin of cofilactin resulted in a similarly bent D-loop due to the K50/E270 linkage (Figure 3E, right), but structural alignment of the filament fragment to cofilactin suggested that cofilin could be sterically tolerated by the cross-linked model filament (Figure 3E). To examine whether our model could stably accommodate cofilin in the structure of a full filament, we constructed 11-monomer “infinite filament” systems. We launched four atomistic 50-nanosecond MD equilibrations based on actin in the experimentally determined cofilactin conformation (Supplementary Figure S2). The timescale was selected to reveal the difference in filament stability at early times. In two simulations (+/− cofilin), we rebuilt the filaments entirely from cross-linked dimers (Supplementary Figure S2B,D) and constructed two more (+/− cofilin) uncross-linked systems as controls (Supplementary Figure S2A,C).

All systems decreased in total actin-actin longitudinal contact surface area over time (Supplementary Figure S2E) as cofilin replaced the initially artificial D-loop contacts. While starting from nearly identical initial contact areas, the covalently modified filaments, either with or without cofilin, had ~20% reduced longitudinal and ~10–20% reduced lateral contact surface area with respect to their un-cross-linked counterparts (Supplementary Figure S2E) at the end of the equilibration. This observation suggests that the K50/E270 cross-linking affects the cofilactin contacts beyond the rearrangements found in normal cofilactin filament.

Despite the observed weakening in protomer-protomer contacts within actin filament, we found similar stability (as reported by backbone root-mean-square deviation (RMSD) of actins only) for cofilactin, cofilactin composed of ACD-cross-linked dimers, and for F-actin in the cofilactin conformation with cofilins removed (Supplementary Figure S2F). This illustrates that either cross-linking actin protomers in cofilactin or removing cofilin is insufficient to destabilize cofilactin at this timescale. In contrast, the cross-linked filament without support from cofilin had consistently higher backbone RMSD (by 2–3 Å). Combined, these data support our experimental observations that the disruptions caused by the cross-link can be offset by cofilin.

### 2.3. Linear and Cyclic AOs Inhibit Similarly Formin- and Ena/VASP-Driven Actin Assembly

**Figure 4:**
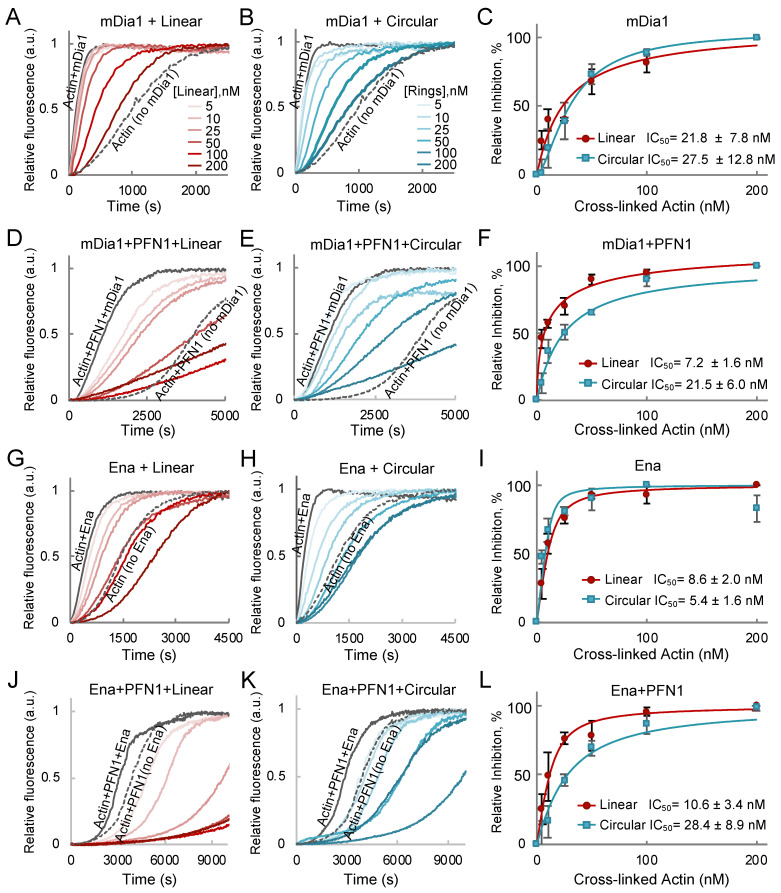
Inhibition of mDia1- and Ena/VASP-controlled actin polymerization by linear and circular actin oligomers in bulk pyrenyl-actin assays. (**A**–**F**) Effects of linear (**A**,**D**) and circular (**B**,**E**) actin oligomers on mDia1-controlled actin polymerization in the absence (**A**–**C**) or presence (**D**–**F**) of PFN1. (**G**–**L**) Effects of linear (**G**,**H**) and circular (**J**,**K**) actin oligomers on Ena/VASP-controlled actin polymerization in the absence (**G**–**I**) or presence (**J**–**L**) of PFN1. (**C**,**F**,**I**,**L**) Inhibition of mDia1- and Ena/VASP-controlled actin polymerization was assessed by calculating the tangent slope of pyrene fluorescence traces at 50% of maximum polymerization and fitting the obtained data to a binding isotherm.

Formins and Ena/VASP (vasodilator-stimulated phosphoprotein) are two families of actin binding proteins that are potently inhibited by AOs [6]. In their active forms, both proteins contain several actin binding domains, which is the key requirement for the high-affinity multivalent binding-based inhibition mechanism employed by AOs. Formins have several profilin-dependent actin binding sites in the FH1 domains, while the FH2 dimer can bind actin in a profilin-independent manner. We have demonstrated that, although the full inhibitory capacity of AOs is achieved when the profilin-dependent FH1 sites are engaged, a potent inhibition can still be achieved in the absence of profilin or FH1 domains [6]. By comparing the inhibitory effects of linear and cyclic AOs, we found that both are equally effective in the absence of profilin (Figure 4A–F). Addition of profilin potentiates the inhibition by linear oligomers by about three-fold (IC_50_ 7 and 22 nM with and without profilin, respectively), but has little effect on inhibition by cyclic AOs (Figure 4C,F).

To compare the effects of linear and cyclic oligomers on actin polymerization by Ena/VASP family of actin organizers, we explored the inhibition of EnaΔL—a Drosophila orthologue of VASP lacking a poorly conserved linker of unknown function [31]. Similar to formins, Ena contains profilin-independent WH2-like G- and F-actin binding domains (GAB and FAB) and poly-proline core profilin binding domains [32]. We found that both types of oligomers inhibited EnaΔL similarly in the absence of profilin, apparently via binding to GAB and FAB domains of EnaΔL tetramers (Figure 4G–I). Notably, the oligomers inhibited Ena-controlled actin polymerization to rates much slower than those demonstrated by actin or actin-profilin alone, in agreement with AOs converting Ena into potent capping proteins [5]. The inhibition of EnaΔL by linear oligomers was not affected by profilin, in agreement with negligible contribution of the poly-proline core to the rate of actin polymerization by Ena [31]; in contrast, the inhibition by cyclic AOs was reduced ~five-fold (IC_50_ 28 and 5.4 nM with and without profilin, respectively; Figure 4G–L). A possible explanation for this difference is that profilin binding sites are more exposed on actin subunits of linear as compared to cyclic AOs. We tested this possibility using coarse-grained molecular dynamics simulations.

### 2.4. Coarse-Grained Simulations Predict Flexibility in AO Structure While Confirming that Profilin Binding Sites Stay Consistently Exposed

To address differences between AO types, we elected to explore AOs from a structural perspective. Structural analysis of AOs by image reconstruction is problematic as populations of ACD-cross-linked actin oligomers are inherently heterogeneous. AO dimers can be purified by cross-linking of K50C and E270Q actin mutants [9,11], but a method to purify homogeneous populations of higher-order N-mers has not been established. Furthermore, K50 and E270 belong to two of the most flexible regions of actin (the D-loop and H-plug, respectively [33], which impart high rotational freedom around the cross-linked residues in AOs, as evidenced by the stark contrast in the two available crystal structures of AOs (PDBs: 3CJB and 3CJC). To overcome these limitations, we elected to simulate the shortest cyclic AOs (i.e., tetramers) and their linear counterparts according to the Kim and Hummer (KH) coarse-grained model [34,35]. The KH model uses residue-level potentials and assumes rigid-body domains yet accurately reproduces binding affinities of transient encounter complexes [35]. This model has been successfully used to replicate aspects of actin nucleation and polymerization in silico [36] and to study transfer of profilin-actin by the FH1 domain of formin [37]. Since the cross-link between actin protomers geometrically reduces the accessible conformation space, we reasoned that coarse-grained simulations would reveal the most likely structures of linear and cyclic AOs.

**Figure 5:**
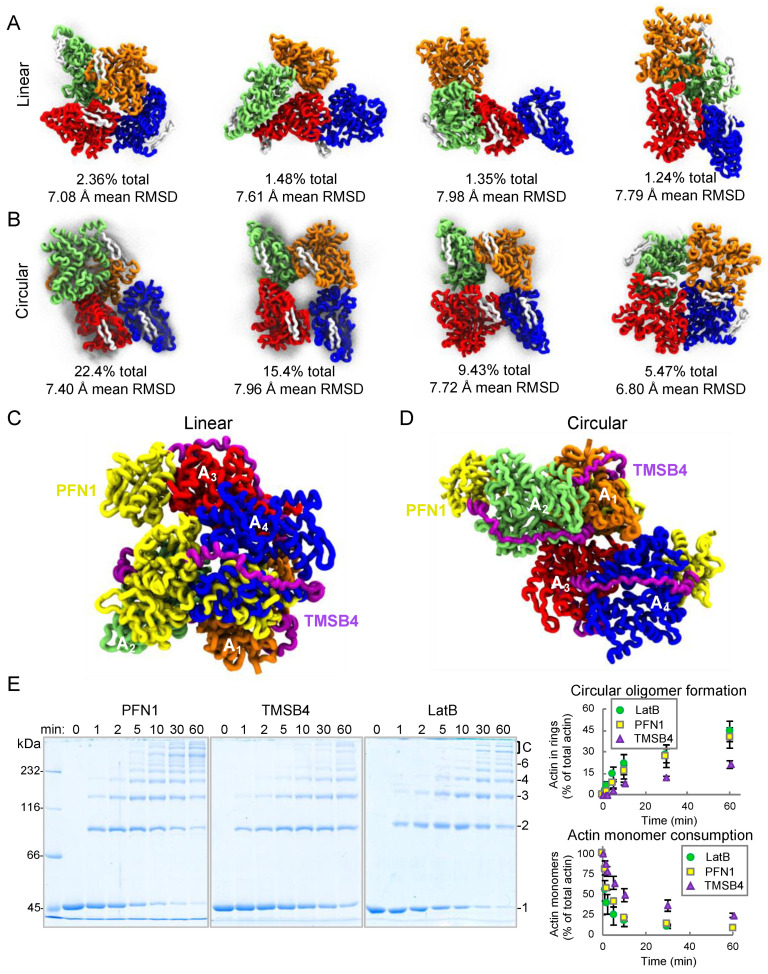
Typical structures of tetrameric AOs generated in coarse-grained molecular dynamics. (**A**–**D**) The four most common structural motifs identified in clustering analysis of linear (**A**) and circular (**B**) tetrameric AOs in coarse-grained molecular dynamics simulations. Percentages correspond to the fraction of structures that belong to the shown cluster, while RMSDs are average among each pair of structures in that cluster. Each transparent gray point corresponds to an alpha carbon from a cluster member, and all alpha carbons are shown. To facilitate comparison, the same hairpin-like region (residues 236–252) is colored white throughout the subunits, and red/blue protomers are always oriented similarly. For clarity, flexible regions are omitted in these representations. Alignment of thymosin-β4 (TMSB4; purple) and profilin (PFN1; yellow) to each actin protomer (**A_1_**–**A_4_**) in the tetrameric linear (**C**) and circular (**D**) AOs. (**E**) Actin cross-linking by ACD was assessed in the presence of latrunculin B (LatB) or G-actin binding proteins PFN1 and TMSB4 added at equimolar concentrations to actin. Uncropped SDS-polyacrylamide gels are shown on the Supplementary Figure S1B. The formation of circular AOs and consumption of actin monomers in the cross-linking reactions were quantified by densitometry.

To enhance conformational sampling, we used replica exchange molecular dynamics (REMD) [38,39] with 28 replicas spanning temperature range 195–368 K (based on [40]). In this setup, any given replica takes a random walk among temperatures in our list, allowing the system to avoid local minima along the potential energy landscape. We combined our 1-µs equilibrations and performed Quality Threshold trajectory clustering analysis [41] at T = 301.2 K to identify the most frequent structural states (Figure 5A–D, Supplementary Figure S3). We evaluated the availability of binding sites at each frame by aligning actin binding proteins and considering two carbons within 3.0 Å to be clashing. We tested binding site exposure for the WH2-motif and two major G-actin binding proteins—profilin (PFN1) and thymosin-β4 (TMSB4). We found that only 18.7% of actin subunits are accessible to TMSB4 in linear and 45.8% in cyclic oligomers. The frequently buried TMSB4 binding sites explain the ~2.5-fold decrease in the ACD cross-linking rate of the TMSB4-actin complex as compared to actin in complex with latrunculin B (Figure 5E), a well-studied G-actin sequestering factor which did not impose steric clashes in any of the configurations.

Profilin can be sterically accommodated in the majority of AO binding sites: 64.4 and 73.2% for structures of linear and cyclic AOs, respectively. This correlates with profilactin being a better substrate for cross-linking than TMSB4-actin complex (Figure 5E). Supporting the strong potential of AOs to inhibit Ena (Figure 4G–L), our simulations revealed that WH2-motif binding sites were constitutively exposed at 96.0 and 99.9% for linear and cyclic AOs, respectively, which is consistent with effective binding to the WH2-like GAB and FAB domains of Ena. As anticipated, we observed higher conformational variability by linear AOs, as evidenced by substantially smaller cluster sizes and the higher average RMSDs across members of each cluster (Supplementary Figure S3).

The higher exposure of profilin binding sites in cyclic AOs does not support our initial tentative explanation of their weaker inhibitory properties in the presence of profilin. It is possible, therefore, that linear AOs are better inhibitors of mDia1 and Ena due to their higher heterogeneity and configurational entropy. The more heterogenous (2 to ~10 protomers) and extended linear AOs may be more geometrically favorable for binding to polyproline regions of mDia1 and Ena than smaller (4–6 protomers) and tightly-packed cyclic AOs. Linear AOs explore a much larger configuration space (Supplementary Figure S3), containing many structures that may be more amenable to high-occupancy binding.

In this study, we discovered and characterized a new cyclic species of oligomeric actin cross-linked by ACD. We showed that AO rings are produced in cells at late stages of ACD toxicity and reproduced them in a test tube. The circular oligomers are similar to the previously reported linear AOs in their in vitro capacity to inhibit the activity of actin assembly factors. We modeled incorporation of AOs into filaments upon stabilization by phalloidin or cofilin and detailed mechanisms by which the stabilization occurs. Atomistic simulations suggested that F-actin can accommodate a D-loop to H-plug covalent cross-link and implied that AO dimers may operate by interrupting both lateral and longitudinal F-actin contacts. In contrast to linear AOs, their cyclic counterparts cannot form filaments in the presence of cofilin, possibly reflecting a lower potential of the host cell to neutralize them. We found that the linear and circular oligomers modeled via coarse-grained molecular dynamics equilibrations expose multiple binding sites for profilin and WH2-motifs, corroborating their high potential to inhibit major F-actin organizing proteins via multivalent interactions. Our observations expand the growing picture of ACD toxicity by clarifying how a low-abundance enzyme can convert a ubiquitous protein into a secondary toxin and indirectly poison regulation in the host cell.

## 3. Materials and Methods

### 3.1. Cell Culture

U2OS, HeLa, and IEC-18 cells were cultured in Dulbecco’s Modified Eagle Medium (DMEM; Thermo Fisher Scientific, Waltham, MA, USA) supplemented with 10% fetal bovine serum (FBS), l-glutamine, and penicillin-streptomycin at 37 °C, 5% CO_2_, and confirmed to be mycoplasma-negative using PCR-based mycoplasma detection analysis [42].

### 3.2. Intracellular Actin Cross-Linking by ACD and Western Blotting

The Anthrax toxin (Atx) translocation system from *Bacillus anthracis* was utilized for the intracellular delivery of ACD [43,44]. Cells were treated with ACD fused with the N-terminal fragment of lethal toxin LF_N_ (LF_N_ACD) pre-mixed with protective antigen (PA; final concentrations: 1 nM LF_N_ACD, 2.5 nM PA). Accumulation of ACD-cross-linked actin species in whole cell lysates was monitored by immunoblotting with pan-actin antibody ACTN05(C4) (1:1000; Thermo Fisher Scientific, Waltham, MA, USA) followed by anti-mouse antibody conjugated with horseradish peroxidase (HRP; 1:10000; Sigma-Aldrich, St. Louis, MO, USA); signal was detected using chemiluminescent HRP substrate WesternBright Sirius (Advansta, Menlo Park, CA, USA) in an Omega Lum G imager (Aplegen, San Francisco, CA, USA).

### 3.3. Protein Purification

*B. anthracis* PA was purified as previously described [45]. Briefly, PA was expressed in BL21(DE3) by isopropyl β-D-1-thiogalactopyranoside (IPTG) induction for 4 h at 30 °C and purified from the periplasmic fraction on DE52 anion exchange resin (Sigma-Aldrich, St. Louis, MO, USA) followed by fractionation by size-exclusion chromatography.

LF_N_ fusions of ACD from *V. cholerae* (LF_N_ACD*_Vc_*) was purified as reported [46,47]. Expression in BL21(DE3)pLysS was induced by IPTG overnight at 15 °C and the protein was purified using TALON metal affinity resin (Clontech, Mountain View, CA, USA) according to the manufacturer instructions.

Skeletal actin was prepared from acetone powder of rabbit skeletal muscle (Pel-Freeze Biologicals, Brown Deer, WI, USA) as previously described, using G-buffer (5 mM Tris-HCl, pH 8.0, 0.2 mM CaCl_2_, 0.2 mM ATP, 5 mM β-mercaptoethanol [β-ME]) and multiple rounds of polymerization and depolymerization [48]. Pyrene labeling was performed as reported [5,6].

ACD from *V. cholerae* (ACD*_Vc_*) and thermolabile ACD from *A. hydrophila* (ACD*_Ah_*) were purified as previously published [49]. Expression in BL21(DE3)pLysS was induced by IPTG (overnight at 15 °C) and ACD constructs were purified using TALON metal affinity resin by standard procedure.

For pyrene-actin polymerization experiments, linear ACD-cross-linked actin oligomers were prepared by mixing 5 µM G-actin with 10 nM ACD*_Ah_* in G-buffer supplemented by 2 mM MgCl_2_ at 10 °C for 25 min. To favor circular oligomer formation, ACD*_Ah_* concentration was increased to 200 nM and the reaction was allowed to proceed at room temperature for 1 h. In both cases, the cross-linking reactions were terminated by heat-inactivation of ACD*_Ah_* at 42 °C for 30 min. Actin was then concentrated ~5–10 fold in a spin concentrator (Sartotius, Göttingen, Germany). To polymerize uncross-linked actin, the concentration of MgCl_2_ was increased to 3 mM and the reactions were incubated at 25 °C for 30 min. F-actin was pelleted by ultracentrifugation at 90,000 rpm for 30 min at 4 °C in a TLA100 rotor (Beckman Coulter, Brea, CA, USA). The supernatants were supplemented with 1 mM ATP and the concentration of monomeric actin equivalents in cross-linked species was determined by Bradford assay (Bio-Rad Laboratories, Hercules, CA, USA) using monomeric actin as a standard. Inactivation of ACD and removal of uncross-linked actin were confirmed as described [6]. ACD-cross-linked actin oligomers and rings were stored on ice and used within 7 days.

For mass spectrometry experiments, actin oligomers were prepared by mixing 20 µM G-actin with 200 nM ACD*_Ah_* in a reaction buffer (5 mM 4-(2-hydroxyethyl)-1-piperazineethanesulfonic acid (HEPES), pH 7.5, 0.2 mM ATP, 1 mM MgCl_2_) on ice overnight. Linear oligomers were then polymerized in the presence of either phalloidin (PHD, Millipore, Burlington, MA, USA) or cofilin at 1:1 molar ratio to actin and separated from cyclic oligomers by pelleting as above.

Tag-less human cofilin 2 (CFL2) was purified as described previously [50,51]. Briefly, CFL2 expressed in *E. coli* BL21-CodonPlus(DE3) (Agilent Technologies, Santa Clara, CA, USA) under IPTG induction (overnight at 25 °C) was purified by sequential anion and cation exchange chromatography: DE52 (DEAE cellulose, Sigma) and SP-sepharose (Sigma-Aldrich, St. Louis, MO, USA) columns connected sequentially. The protein was eluted from SP-sepharose column with a 50 to 500 mM NaCl gradient and further purified using size-exclusion chromatography in a buffer containing 10 mM piperazine-*N*,*N*’-bis(2-ethanesulfonic acid) (PIPES), pH 6.8, 25 mM KCl, 1 mM dithiothreitol (DTT), 0.4 mM ethylene glycol-bis(β-aminoethyl ether)-*N*,*N*,*N*’,*N*’-tetraacetic acid (EGTA), 0.1 mM phenylmethylsulfonyl fluoride (PMSF).

Tag-less human thymosin β4 (TMSB4) was expressed in *E. coli* BL21(DE3) and purified on HiTrap SP HP ion exchange column as previously described [52]. TMSB4 was stored in a buffer containing 5 mM Tris-HCl, pH 7.9, 40 mM KCl, 0.2 mM DTT, 0.1 mM PMSF, 0.01% sodium azide.

A mouse formin mDia1 construct containing FH1, FH2, and DAD domains (residues 552–1255 including all 14 polyproline stretches of FH1 domain of mDia1 protein) was prepared as described previously and stored in a buffer containing 20 mM Tris-HCl, pH 8.5, 50 mM NaCl, 50% glycerol, 1 mM DTT, 0.1 PMSF at −20 °C [6,53].

A recombinant construct of *Drosophila melanogaster* Ena protein lacking a linker region (a.a. 113–299) Ena∆L was prepared as previously described [31]. Briefly, Ena∆L was purified using TALON cobalt resin (Clontech, Mountain View, CA, USA) in buffer A (50 mM NaH_2_PO_4_, 500 mM NaCl, 10% glycerol, 10 mM imidazole, and 0.5 mM PMSF), eluted in buffer B (buffer A supplemented to 250 mM imidazole), and stored in buffer C (20 mM HEPES, 1 mM ethylenediaminetetraacetic acid (EDTA), 200 mM KCl, 5 mM β-ME, 10% glycerol, and 0.1 mM PMSF).

Human profilin 1 (PFN1) was purified as previously described [6] by binding to a poly-l-proline sepharose resin, followed by elution under denaturing conditions and dialysis against three buffer changes of storage buffer (2 mM Tris-HCl, pH 8.0, 0.2 mM EGTA, 1 mM DTT, 0.1 mM PMSF).

### 3.4. Transmission Electron Microscopy

For imaging of ACD-cross-linked circular actin oligomers, samples were applied to carbon-coated grids (Ted Pella, Redding, CA, USA) for 60 s and negatively stained with 1% (*w*/*v*) uranyl acetate. Grids were examined in a Tecnai-12 (Philips, Amsterdam, Netherlands) electron microscope at an accelerating voltage of 80 keV and a nominal magnification of 60,000×.

### 3.5. Pyrenyl-Actin Polymerization Assays

Actin polymerization assays were carried out as previously described [5,6]. Briefly, pyrene-labeled, gel-filtered Ca^2+^-actin (5% labeled; 2.5 µM final concentration) was pre-incubated with mDia1 or EnaΔL in the presence or absence of PFN1 and varying concentrations of actin oligomers or actin rings (0–200 nM) in reaction buffer (10 mM 3-(*N*-morpholino)propanesulfonic acid (MOPS), pH 7.0, 0.2 mM ATP, 0.5 mM DTT). Ca^2+^-ATP actin was then converted to Mg^2+^-ATP actin by the addition of 0.066 volumes of switch buffer (150 mM MOPS, pH 7.0, 3 mM ATP, 7.5 mM DTT, 4.5 mM EGTA, 1.5 uM MgCl_2_) and incubation for 2 min. Polymerization was initiated by the addition of 0.33 volumes of initiation buffer (30 mM MOPS, pH 7.0, 0.6 mM ATP, 1.5 mM DTT, 3 mM MgCl_2_, 150 mM KCl) and monitored at λ_ex_ = 365 nm and λ_em_ = 407 nm on the Infinite M1000 Pro plate reader (Tecan US, Inc., Morrisville, NC, USA).

Inhibition of polymerization by linear or circular actin oligomers was assessed by calculating the tangent slope of each pyrene fluorescence trace at 50% (40–60% interval) of maximum polymerization and fitting the obtained data to a binding isotherm [54].

### 3.6. In Vitro Actin Cross-Linking Assays

ACD-catalyzed actin cross-linking was performed as described previously [9] using 5 μM G-actin and 10 nM ACD*_Vc_* or ACD*_Ah_* in the presence or absence of either 5 μM LatB (Millipore, Burlington, MA, USA), 5 μM PFN1, or 5 μM TMSB4. The reactions were initiated by addition of MgCl_2_ (2 mM) and stopped by addition of *N*-ethylmaleimide (1 mM) and boiling the samples in a reducing SDS-sample buffer. The samples were analyzed on 7.5% SDS-polyacrylamide gels. The formation of circular actin oligomers and consumption of actin monomers in the cross-linking reactions was quantified using ImageJ software [55]. When required to enrich circular oligomers, following incubation with ACD, linear oligomers were polymerized in the presence of either phalloidin (Millipore, Burlington, MA, USA) or CFL2 at 1:1 molar ratio to actin and pelleted by ultracentrifugation at 90,000 rpm for 30 min at 4 °C in a TLA100 rotor (Beckman Coulter, Brea, CA, USA). Supernatants (containing circular oligomers) and pellet fractions were resolved on 7.5% and 10% SDS-polyacrylamide gels.

### 3.7. Mass Spectrometry

G-actin (20 μM) was cross-linked by addition of ACD*_Vc_* (10 nM) followed by pelleting either in the absence or presence of CFL2 or PHD as described above. Supernatant fractions were collected and analyzed using mass spectrometry. 1-μL samples were injected onto a self-packed buffer exchange column (P6 polyacrylamide gel, Bio-Rad Laboratories, Hercules, CA, USA) and buffer exchanged into 200 mM ammonium acetate (pH 6.8) at a flow rate of 100 μL/min using a Vanquish UHPLC (Thermo Fisher Scientific, Waltham, MA, USA) [56]. Samples were ionized using a heated electrospray ionization (HESI) source with a spray voltage of 3.75 kV and source temperature of 275 °C. Samples were sprayed into a Q Exactive ultra-high mass range (UHMR) instrument modified with a surface induced dissociation (SID) device (not used in this study) [57]. Mass spectra were collected at 1000 to 16,000 m/z range at 6000 resolution (at 400 m/z). In-source trapping of 200 V and higher-energy collision dissociation (HCD) of 120 V were applied to remove adducts; harsh conditions were considered acceptable because of the covalent nature of the analyte. Acquired spectra were averaged across the elution time of the protein. Deconvolution of the averaged spectrum and mass determination was accomplished manually and/or using UniDec software [58].

### 3.8. Molecular Dynamics Simulations

To simulate the Glu-Lys isopeptide bond in the CHARMM36 force field [59], we modified by analogy the DKAM patch (patch to create D-K AMide bond) from the July 2020 release of the standard C36 distribution. H73 on actin was always methylated by patch MHSD. We extracted the actin dimer from PDB: 3CJC in Chimera [60] and selected appropriate rotamers for K50 and E270 from the Dunbrack 2010 library [61] via Chimera Rotamers. Pre-targeting dimers were aligned to F-like trimers extracted from PDB: 6T1Y (phalloidin) or PDB: 6VAO (cofilin) in Chimera MatchMaker. The cofilin D-loop was copied from 6T1Y after local alignment. ADP was replaced with ATP and Ca^2+^ with Mg^2+^. Both phalloidin and cofilin were removed before the targeting procedure. No forces were applied to any D-loop (residues 39 to 51) or H-plug (residues 263 to 273); forces were applied to all other protein elements. Post-targeting, cross-linked dimers from the cofilin system were replicated and aligned to the 6VAO to create full 11-mer filaments, which were again minimized (see below) before production simulations. The simulation box was selected to allow the barbed end to interact with the pointed end via periodic image.

All systems were prepared in VMD [62] version 1.9.4 according to the following procedure: (1) PSF structure preparation through VMD AutoPSF (psfgen and TopoTools 1.8 [63]); (2) solvation in rectangular boxes by VMD Solvate to guarantee at least 1.5 nm separation from any orientation of the periodic image; (3) sequential ionization to neutralize system charge and reach 100 mM KCl, 10 mM NaCl, and 1 mM MgCl_2_. Final systems contained ~500,000 atoms; 50 ns equilibration or targeting runs were integrated with a 2 fs timestep and preceded by 10,000 steps of minimization and 200 ps of fixed-backbone equilibration in NAMD 2.12 [64]. Electrostatic interactions were calculated using the Particle Mesh Ewald method and pressure was controlled by the hybrid Nosé-Hoover Langevin piston with target pressure 1 atm, oscillation period 200 fs, and decay time 100 fs. Temperature was modulated around target 310 K using Langevin dynamics with a damping constant of 0.1 ps^−1^. All figures used Tachyon ray tracing [63] and STRIDE secondary structure prediction [65]. Contact surface calculations were done through PyContact [66].

### 3.9. KH Model Coarse Graining

Coarse-grained simulations were carried out using the KH model [34], with the same parameters as in [37], unless otherwise stated. In the KH model, each amino acid is represented with a single bead at the location of the α carbon with pairwise interactions modeled by Lennard-Jones (LJ)-type potentials, for which we used a 3σ cutoff. For electrostatic interactions, we used a screening length of 10 Å, and uniform dielectric constant 80. For this work we implemented KH Model A using continuous piecewise potentials (code available at https://github.com/aah217/KH_LAMMPS) and utilized LAMMPS Version 3 Mar 2020 [67]. Our implicit-solvent systems are comprised of semi-rigid G-actin monomers connected by flexible K50/E270 cross-links, which are approximated by a series of three flexible harmonic bonds of length 3.81 Å. For rigid segments we applied the fix rigid command in LAMMPS, which sums all forces and torques on the individual particles and causes the unit to move together as one body. Beads in flexible regions were connected by harmonic springs with equilibrium length 3.81 Å without angular or dihedral constraints. Our initial tetramer structures were derived by hand alignment of G-actin from a proposed model of ACD bound to two G-actin monomers [68]. To reproduce the flexibility of G-actin, we kept the D-loop (residues 38–52), H-plug (residues 264–272), and N-/C-termini (residues 1–5, 372–375) flexible while treating the rest as a rigid body. To increase rigid-body diffusion, we reduced the mass of our rigid-body systems by a factor equal to the number of residues [37]. Integration was performed with a 0.1-fs timestep for an initial minimization period, and later increased to 10 fs for sampling. Our neighbor lists were updated every 10 steps and Monte Carlo temperature swapping was attempted every 100 steps. Simulations were carried out in the NVT ensemble. We simulated AO rings and oligomers and extracted frames at each temperature using MDAnalysis [69]. Clustering was at 301.2 K via a modified implementation of Quality Threshold clustering [41] with an RMSD cutoff of 13.0 Å. When constructing the pairwise RMSD matrix used in clustering, we ensured that each actin protomer could be aligned to any other protomer. Aligned actin binding molecules for quantifying contacts had PDB IDs 2BTF (profilin) [70], 1ESV (GS1) [71], 4PL7 (TMSB4) [72], and 2A3Z (WASP WH2-motif) [73]. To quantify binding accessibility, we used MDAnalysis to align actin binding proteins (TMSB4, profilin, and WH2) and counted the accessible binding sites by checking whether each actin-binder became closer than 3.0 angstroms to each actin protomer that was not involved in actin binding protein alignment.

## Data Availability

The data from this study are presented within the figures and Supplementary Information and are available on request from the corresponding author. Data generated in the coarse-grained simulations are not hosted online due to the large amount of data generated.

## Supplementary Materials

Supplementary Materials can be found at https://www.mdpi.com/1422-0067/22/2/718/s1.

## Abbreviations

ACD	Actin cross-linking domain
AO	Actin oligomers
Atx	Anthrax toxin
β-ME	β-mercaptoethanol
CFL2	Cofilin 2
DMEM	Dulbecco’s modified Eagle medium
DTT	Dithiothreitol
EDTA	Ethylenediaminetetraacetic acid
EGTA	Ethylene glycol-bis (β-aminoethyl ether)-*N*:*N*,*N*’,*N*’-tetraacetic acid
FAB	F-actin binding domain
FBS	Fetal bovine serum
GAB	G-actin binding domain
GS1	Gelsolin segment 1
HEPES	4-(2-hydroxyethyl)-1-piperazineethanesulfonic acid
HRP	Horseradish peroxidase
IC_50_	Half-maximal inhibitory concentration
IPTG	Isopropyl β-d-1-thiogalactopyranoside
LatB	Latrunculin B
LF_N_	N-terminal fragment of lethal toxin
MARTX	Multifunctional auto-processing repeats-in-toxin
MOPS	3-(*N*-morpholino)propanesulfonic acid
MS	Mass spectrometry
PA	Protective antigen
PFN1	Profilin 1
PHD	Phalloidin
PIPES	Piperazine-*N*,*N*’-bis(2-ethanesulfonic acid)
PMSF	Phenylmethylsulfonyl fluoride
PP	Polyproline
REMD	Replica exchange molecular dynamics
RMSD	Root-mean-square deviation
TMD	Targeted molecular dynamics
TMSB4	Thymosin β4
VASP	Vasodilator-stimulated phosphoprotein
VgrG1	Valine-glycine repeat protein G1
WASP	Wiskott-Aldrich syndrome protein
WH2	WASP-homology motif 2
