# Supplementary material for: Rounding Out the Understanding of ACD Toxicity with the Discovery of Cyclic Forms of Actin Oligomers
Source: Int J Mol Sci. 2021 Jan 13;22(2):718. doi: 10.3390/ijms22020718 (PMC7828245; doi:10.3390/ijms22020718)
Supplement: Supplementary file 1 [file ijms-22-00718-s001.pdf]

Supplementary Information for

Rounding out the understanding of ACD toxicity with the discovery of cyclic forms of actin oligomers

Harper Smith, David B. Heisler, Nick Pinkerton, Elena Kudryashova, Aaron R. Hall, Kelly R. Karch, Andrew Norris, Vicki Wysocki, Marcos Sotomayor, Emil Reisler, Dimitrios Vavylonis, and Dmitri S. Kudryashov

- Supplementary Figures S1-S3
- Supplementary Table S1
- Supplementary Movie Legends

Supplementary Figure S1

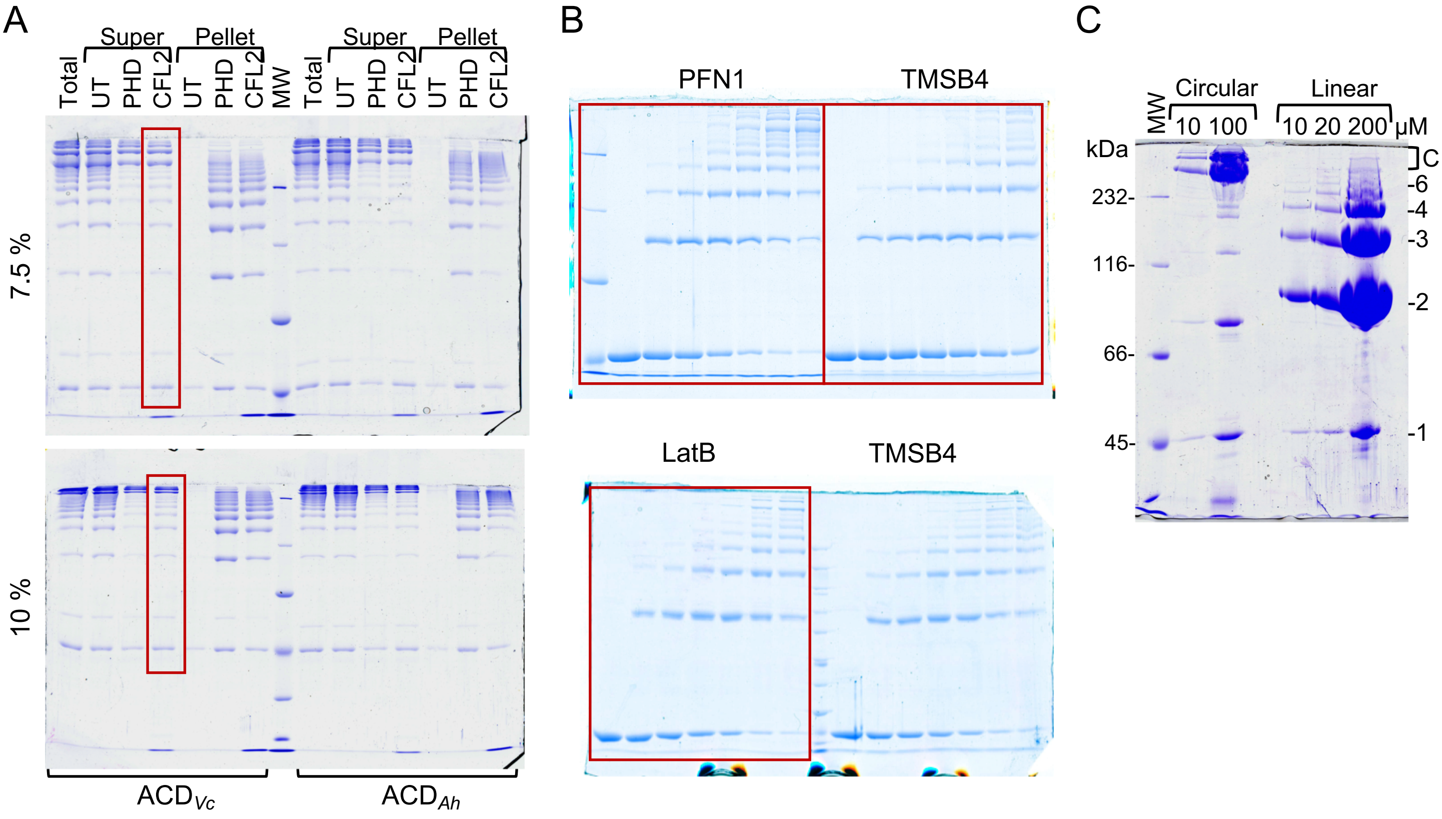

Figure S1. Full-size uncropped gel images.
Boxed areas from (A) and (B) are shown and described in Figures 1B and 5E, respectively.
(C) Representative gel image of circular and linear ACD-cross-linked actin oligomers used in actin pyrene assays presented in Figure 4.

Supplementary Figure S2

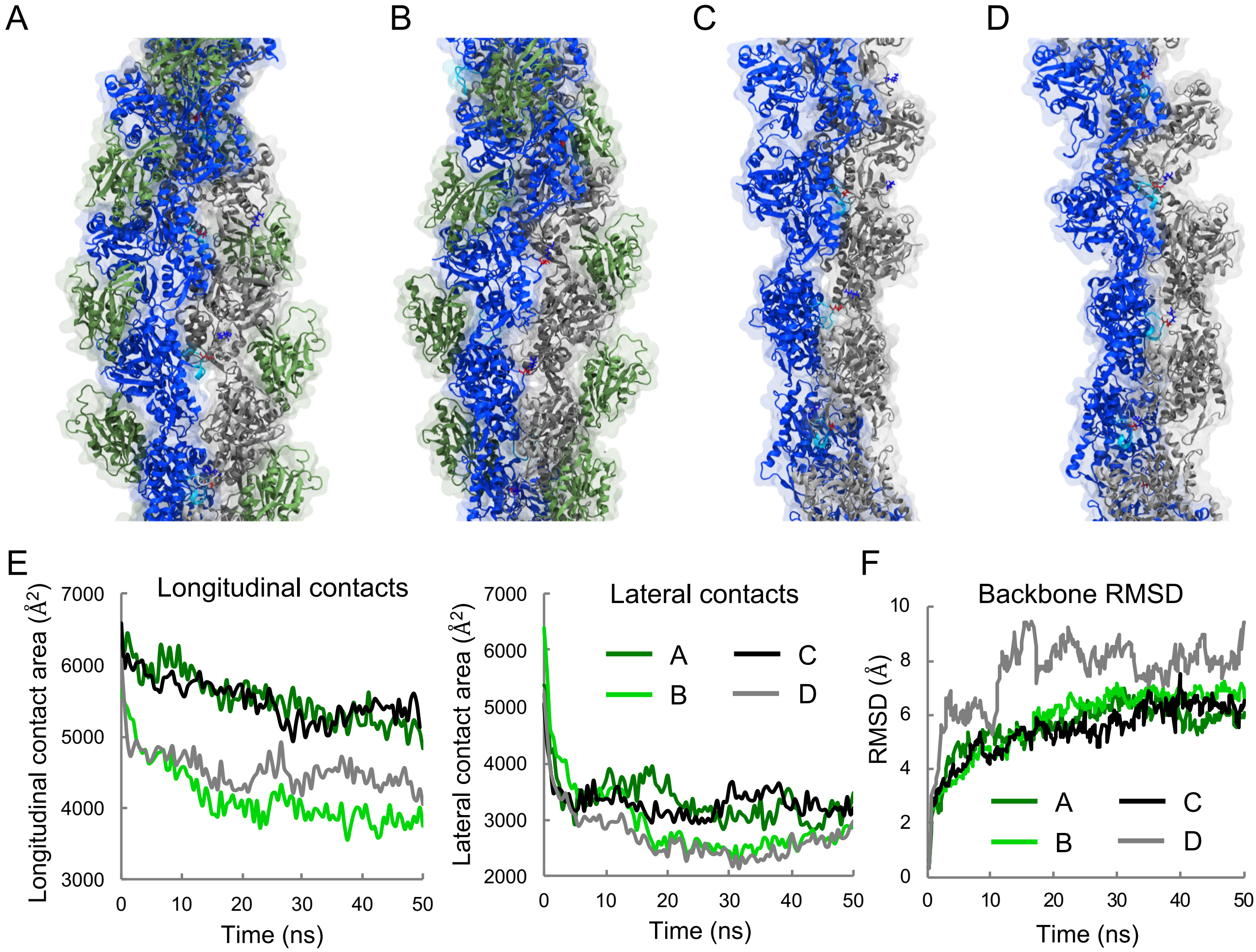

Figure S2. Equilibrations of targeting-derived structures.
Initial structures for 50-ns equilibrations were built from cofilin-actin (cofilactin; PDB: 6VAO). In all cases, coloring is the same as in Figure 3 in the main text: actin subunits are in grey and blue, D-loop (light gray), hydrophobic plug (cyan), K50 (blue sticks), E270 (red sticks), cofilin is in green. (A-D) The post-equilibration structures are shown for cofilactin (A), cofilactin built by alignment with post-targeting ACD-crosslinked dimer (B), cofilactin with cofilins removed (C); and cofilactin with cofilins removed and actins replaced by AO dimers (D). Contact surfaces (E) for structures shown on A-D were calculated by PyContact between each longitudinal pair of protomers and summed (left) and between strands of the filament (right). Backbone RMSDs (F) of the most stable residues in actin for structures shown on A-D were calculated as described in Methods.

Supplementary Figure S3

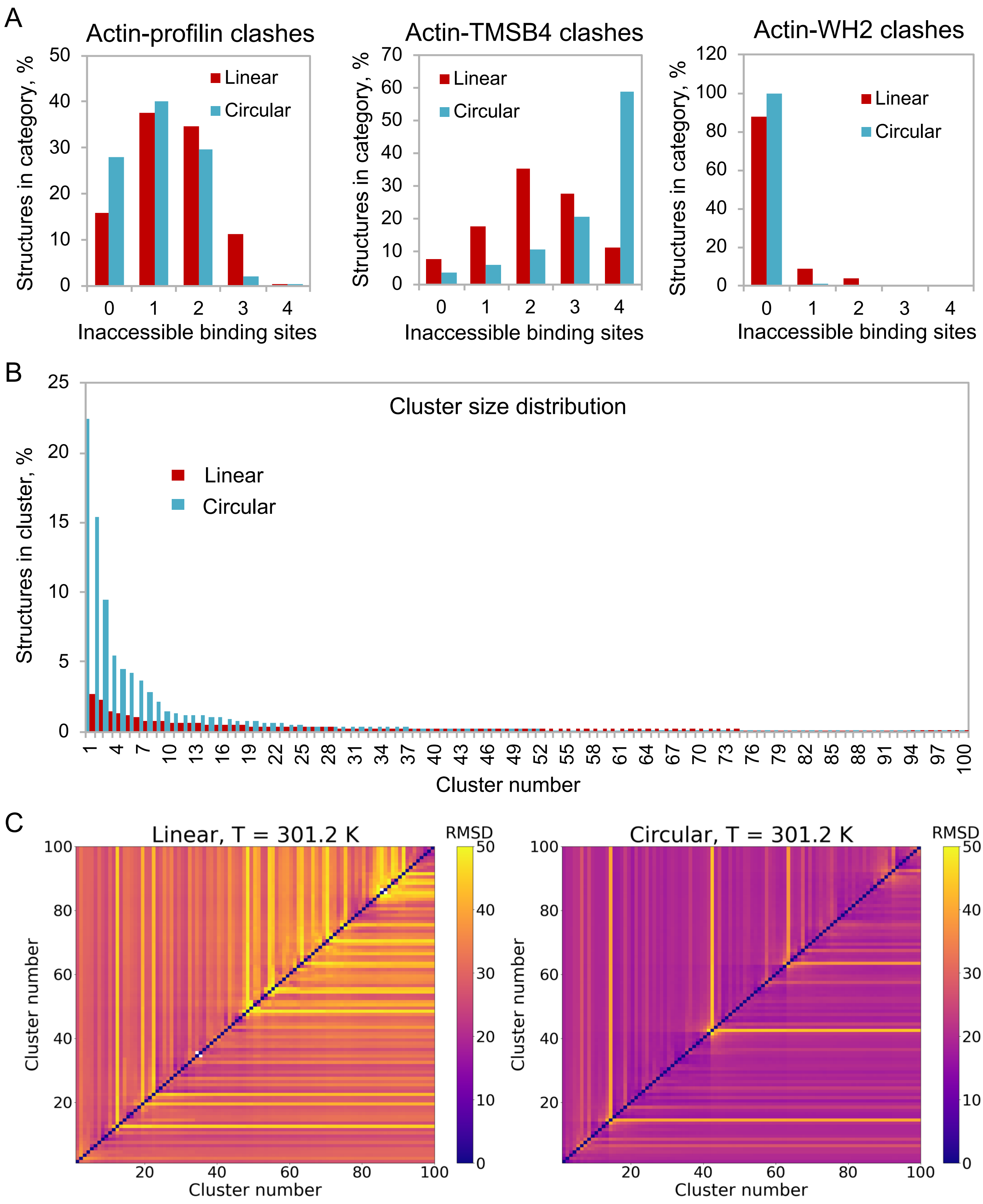

Figure S3. Binding site accessibility and clustering analysis of coarse-grained KH simulations.
(A) Histograms of inaccessible binding sites for actin-profilin (left), actin-TMSB4 (middle), and actin-WH2 (right). Percentages are out of 10,000 frames at temperature T = 301.2 K. For circular and linear AOs, clusters determined by the quality threshold clustering algorithm are shown according to cluster size (B) and average cluster-cluster RMSD (C). Since the circular AOs produce larger clusters (B) and lower RMSDs between clusters (C) compared to linear AOs, they explore a smaller configurational space in our simulations. Only the top 100 clusters are shown, which contain 98.24 and 36.68% of frames for cyclic and linear AOs, respectively. The maximum RMSD cutoff for any two cluster members was set at 13.0 Å. Clusters corresponding to the initial configuration were not considered and, therefore, are not shown.

Supplementary Table S1

Protein	Expected mass (Da)	Untreated oligomers (circular and linear)		Circular (enriched by CFL2 pelleting)		Circular (enriched by PHD pelleting)	
		Observed mass	Δm	Observed mass	Δm	Observed mass	Δm
CFL2	16917	18210	1293	18210	1293	-	-
ACD	54427	53940	487	53940	487	53940	487
dimer	83633	84970	1337	84920	1287	86280	1310
trimer	125450	127470	2020	127490	2040	-	-
tetramer	167267	169840	2573	169770	2503	169780	2513
pentamer	209084	212330	3247	212300	3217	212330	3247
hexamer	250900	254920	4020	254850	3950	254800	3900
heptamer	292717	297380	4663	-	-	297170	4453
octamer	334534	339920	5386	-	-	-	-
nonamer	376350	382420	6070	-	-	-	-

Table S1. Expected and observed masses of proteins detected in native mass spectrometry experiments.
The expected masses were calculated based on the protein sequences. The observed masses calculated using UniDec are 1-4 kDa larger than the expected masses (Δm), most likely due to ATP binding (in case of actin; 507 Da per ATP molecule), salt adducts and/or incomplete desolvation of the complexes.

Supplementary Movie Legends

Supplementary Movie S1. Targeting of ACD-cross-linked dimer to phalloidin-actin. 25-frame/second simulations described in Figure 3B. Each frame corresponds to 0.2 ns. The target structure is transparent and the purple/cyan protomers are extracted from PDB: 3CJC while the green protomer is from phalloidin-actin (PDB: 6T1Y).

Supplementary Movie S2. Targeting of ACD-cross-linked dimer to cofilin-actin. 25-frame/second simulations described in Figure 3C. Each frame corresponds to 0.2 ns. The target structure is transparent and the purple/cyan protomers are extracted from PDB: 3CJC while the green protomer is from cofilin-actin (PDB: 6VAO).
